# Behavioral Prevention, Treatment, and Rehabilitation of Using Western and Chinese Medicines or Herbal Products among the Public in Response to COVID-19 in Hong Kong: A Cross-Sectional Study

**DOI:** 10.1155/2023/5637720

**Published:** 2023-08-30

**Authors:** Siu Kan Law, Dawn Ching Tung Au, Wesley Yeuk Lung Chow, Chung Hang Poon, Kylie Ka Ching Chow, Zhongzhen Zhao, Shun Wan Chan, Yanping Wang, Saimei Li

**Affiliations:** ^1^Faculty of Science and Technology, The Technological and Higher Education Institute of Hong Kong, Tsing Yi, New Territories, Hong Kong; ^2^Hong Kong Chinese Medicine Pharmacists Association, San Po Kong, Hong Kong; ^3^The First Affiliated Hospital of Guangzhou University of Chinese Medicine, Guangzhou, Guangdong, China; ^4^School of Chinese Medicine, Hong Kong Baptist Univesity, Kowloon Tong, Hong Kong; ^5^School of Nursing and Health Studies, Hong Kong Metropolitan University, Ho Man Tin, Kowloon, Hong Kong

## Abstract

The coronavirus disease 2019 (COVID-19) pandemic occurred in Hong Kong for more than two years. This article conducted a cross-sectional study for participants to investigate the behavioral prevention, treatment, and rehabilitation of using Western medicines or herbal products for COVID-19 in Hong Kong. A questionnaire was designed and performed over 2 weeks from 1 May to 15 May 2022. It consisted of five parts with around 20 questions conducted including sociodemographic information, prevention, treatment, rehabilitation of COVID-19, and also the sources of information. The pattern usage of Chinese or Western medicines for COVID-19 was studied after data collection. 318 people participated in this survey, and only 311 were qualified. The sociodemographic information, e.g., personal educational level, and behavior for the prevention of COVID-19, which included wearing masks (98.7%), using alcohol hand sanitizer (83.0%), washing hands frequently (82.4%), avoiding crowds (53.1%), and staying home more often (50.6%). Western medicines, such as antipyretic drugs, antitussive drugs, and pain reliever drugs, whilst Chinese medicines, such as Lianhua Qingwen Jiaonang, Huoxiang Zhengqi San or Wan, and Nin Jiom Pei Pa Koa, were most commonly used in the treatment and rehabilitation periods of COVID-19. Herbal products, including lemon, honey, ginger, and herbal tea, were used as a daily diet to fight against COVID-19. Based on the result findings, Chinese medicines or herbal products were used during the COVID-19 pandemic, but most of the participants used an unknown Chinese medicine practitioner's prescription and self-administered Chinese medicine. The pattern of Chinese medicines and Western medicines' usage in the prevention, treatment, and rehabilitation of COVID-19 was also investigated; this showed a statistically significant association between the variables according to gender, age, and Chinese or Western medicines for further investigation.

## 1. Introduction

In the late December 2019, there was an outbreak of pneumonia with unknown etiology emerged in Wuhan of Hubei Province, China. Chinese scientists identified that it was coronavirus and named it “severe acute respiratory syndrome coronavirus-2” (SARS-CoV-2) [1, 2]. The World Health Organization (WHO) called this coronavirus disease act COVID-19 and declared it a public health emergency of international concern on 30th January 2020 [3]. COVID-19 was defined as a global pandemic, which potentially required a coordinated international response among 25 countries worldwide, including Southeast Asia and Europe, such as Thailand, Republic of Korea, Japan, Philippines, Vietnam, United States, and United Kingdom [4]. As of 16th May 2022, there have been 519,105,112 confirmed cases of COVID-19, and 6,266,324 deaths globally documented by WHO [5].

The first confirmed case in Hong Kong was recorded on 23rd January 2020 and announced by the Center for Health Protection (CHP) [6]. Hong Kong recorded 1,208,740 confirmed cases of COVID-19 and 9361 deaths as of 16 May 2022 [7]. Compared with other countries around the world, Hong Kong had a relatively low number of COVID-19 confirmed cases despite being an international travel hub and its proximity to Wuhan, China. Since Hong Kong's government implemented multiple measures before the first case of COVID-19 was diagnosed, Hong Kong also focused on primary care including the usage of public primary care clinics as part of an enhanced surveillance program together with accident and emergency departments, as well as triaging patients with suspected infections to hospitals [8]. It was a multipronged infection control to achieve zero COVID-19 infection [9, 10]. Nevertheless, the main challenge to Hong Kong's health system was the rising number of COVID-19 infections and a lack of sufficient hospital-based intervention for the infected COVID-19 patients [11].

Personal behavior is one of the important things for primary care. According to WHO, there is some health advice on personal behavior, such as wearing masks, avoiding crowds, washing hands frequently, using alcohol hand sanitizer, staying home more often, and working at home [12]. Growing evidence has shown that Western medicines and herbal foods or products are the possible methods to prevent, treat, and rehabilitate COVID-19. Complementary and alternative medicines are beneficial for COVID-19 by improving immune system function [13]. Western medicines including vitamin and mineral supplements, e.g., vitamin C, vitamin D, vitamin B, and zinc boost the immune system versus viruses. Paracetamol, antihistamines, antiasthmatics, and ivermectin are greatly reducing the risk of COVID‐19 exacerbation [14]. Herbal foods or products are the common approaches to people for the prevention, treatment, and rehabilitation of COVID-19. In early 2020, traditional Chinese medicines (TCM) such as *Tinospora cordifolia* (Willd.) Miers, *Withania somnifera* (L.) Dunal, *Scutellaria baicalensis* Georgi., and *Curcuma longa* L. are effective during this worrisome coronavirus pandemic, which utilized with over 90% efficacy to inhibit the SARS-CoV-2, cure, or reduce the COVID-19 symptoms [15]. Daily diet or dietary supplements provide a potentially convenient and accessible way for COVID-19 recovery that shortens the rehabilitation period [16].

According to a previous investigation, people buy blindly and herd medicines because of the panic and fear surrounding the pandemic combined with misinformation from social and digital media, leading to global medicine shortages, and tend to self-medication for combating the COVID-19 infections [17]. Meanwhile, Bangladesh applied high preventive measures for COVID-19 by the public, and there were a considerable number of participants taking preventive medicines and herbal foods/products [18]. Is this same as the Hong Kong situation? Thus, its survey-based research started with the behavioral prevention of COVID-19, which aimed to explore the prevalence and patterns of using Western medicines or herbal products used among Hong Kong residents during the COVID-19 pandemic for three stages including prevention, treatment, and rehabilitation periods. The pattern of Chinese medicines and Western medicines' usage in the prevention, treatment, and rehabilitation of COVID-19 based on age and gender, as well as Chinese and Western medicines, was investigated independently and analyzed.

## 2. Methods

### 2.1. Study Design and Sampling

This was a cross-sectional survey study performed over 2 weeks from 1st May to 15th May 2022 using a self-administered questionnaire. The study was approved by the Survey and Behavioral Research Ethics Committee of the Faculty of Science and Technology, The Technological and Higher Education Institute of Hong Kong, Tsing Yi, New Territories, Hong Kong, China.

### 2.2. Participants

Participants must be aged 18 years or above who can be eligible to participate in this survey but are not limited to their sociodemographic backgrounds. Data collection was conducted both in-person and online using purposive sampling to recruit participants. An online survey was conducted using a Google questionnaire on social media platforms, including Facebook, WhatsApp, WeChat, and E-mail, while an in-person survey was done by Registered Chinese Medicine Practitioner face-to-face with participants. They were asked for written consent before taking the survey, either online or in-person modes. If the participant was chosen for the online survey, they must click the “Yes” button after written consent to start the survey, as well as proceed to the next section until the questionnaire is completed.

### 2.3. Questionnaire

The questionnaire was developed by the Chinese Medicinal Pharmacy research team from The Technological and Higher Education Institute of Hong Kong and the Hong Kong Chinese Medicine Pharmacists Association. Its contents consisted of five parts with around 20 questions (*S1 File. Questionnaire for English and Chinese version*). This is an open-end questionnaire. Participants were only required to fill out the multiple choices and write short answers for preventing any mistakes or data redundancy. The major concepts were compared to the differences in participants' usage of traditional Chinese medicines or Western medicines for three stages including prevention, treatment, and rehabilitation periods, which were supplementary with the sociodemographic and sources of information.

  Part I: Sociodemographic information, such as gender, education, place of residence, and presence of chronic disease;
  Part II: Participants' behavior for personal protection, and the usage of Western medicines and herbal foods or products for prevention of COVID-19. Participants were asked whether they got any experience with the COVID-19 relating symptoms or not before going to part III. If not, participants were jumped to part V;
  Part III: Participant's usage of Chinese medicines or Western medicines for the treatment of COVID-19. The types of Chinese medicines include Chinese medicine practitioner prescription, folk prescription, self-administered Chinese medicine, and daily diet, whilst Western medicines include prescription drugs, self-purchasing medicines, and self-medications;
  Part IV: Participants' usage of Chinese medicines or Western medicines for the rehabilitation of COVID-19;
  Part V: Sources of information for the Chinese medicines or Western medicines on COVID-19.
  Part VI: The pattern of Chinese medicines and Western medicines in the prevention, treatment, and rehabilitation of COVID-19 usage based on (i) *age* and (ii) *gender*, as well as (iii) *Chinese and Western medicines* (generated and analyzed after data collections from Parts I to IV).

### 2.4. Statistical Analysis

The questionnaire was collected from the Google website, and data were downloaded in the form of an Office Excel spreadsheet, which was used to sort the necessary data and eliminate any invalid questionnaires. Reliable data were sorted and imported into SPSS 24.0 software for statistical analysis. The chi-square test and post hoc test (Bonferonni correction) were used to test the questionnaire's statistical hypothesis and differences that checked with a significant level (*α* = 0.05) [19].

## 3. Results

### 3.1. Part I: Participants' Sociodemographic Information

A total of 318 people participated in the survey, which included gender, education, place of residence, and presence of chronic disease. There were females (54.4%) and males (45.6%) ranging from 18–27 (15.7%), 28–37 (37.4%), 38–47 (15.7%), 48–57 (15.4%), 58–67 (10.4%), 68–77 (4.1%), and 78 (1.3%) above years old. An educational level was between primary school or below (5.0%), secondary school (32.4%), high diploma or associate degree (15.7%), degree (34.3%), master's degree (10.4%), and doctoral degree (2.2%). The place of residence was Hong Kong Island (10.7%), Kowloon (27.0%), New Territories (58.2%), Islands District (2.8%), and unknown (1.3%). Around 19.2% of participants were present at least with an illness, such as diabetes (27.9%), cardiovascular (19.1%), cancer (11.8%), gout (5.9%), respiratory (4.4%), musculoskeletal (4.4%), and others diseases (45.6%) (Table 1).

### 3.2. Part II: Prevention of COVID-19

**Figure 1:**
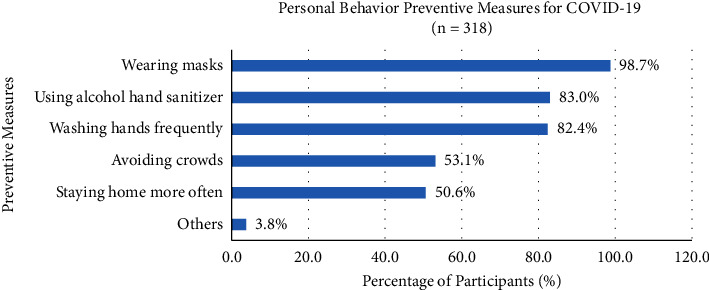
Participants' behavior preventive measures for COVID-19.

**Figure 2:**
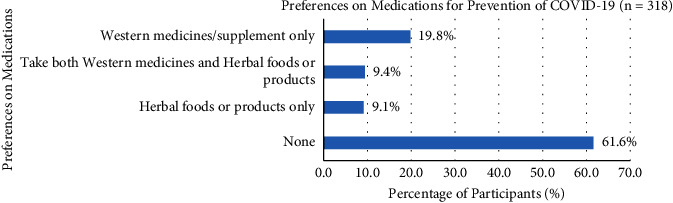
Participants' preferences for medications in the prevention of COVID-19.

Five guidelines on the personal behavior preventive measures for COVID-19 consisted of wearing masks (98.7%), using alcohol hand sanitizer (83.0%), washing hands frequently (82.4%), avoiding crowds (53.1%), staying home more often (50.6%), and other (3.8%) preventions (Figure 1). The preferences of participants' medication used, such as Western medicines or supplements only (19.8%), taking both Western medicines and herbal foods or products (9.4%), herbal foods or products only (9.1%), and more than 61.6% of participants without using Western or Chinese medicine for the prevention of COVID-19 (Figure 2). Western medicines or supplements included vitamin supplements, e.g., C, D, B, and multivitamins (56.7%), zinc (3.3%), and paracetamol (3.3%), were commonly chosen by the participants, but with no antihistamines, arsenicum album, antiasthmatics, ivermectin, and other types (36.7%) of Western medicines or supplements, whilst herbal soup (10.3%) was usually used as herbal foods or products by the participants for the prevention of COVID-19.

### 3.3. Part III: Treatment of COVID-19

**Figure 3:**
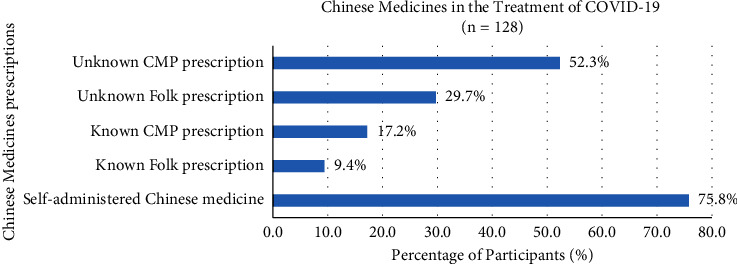
Chinese medicine prescriptions in the treatment of COVID-19.

**Figure 4:**
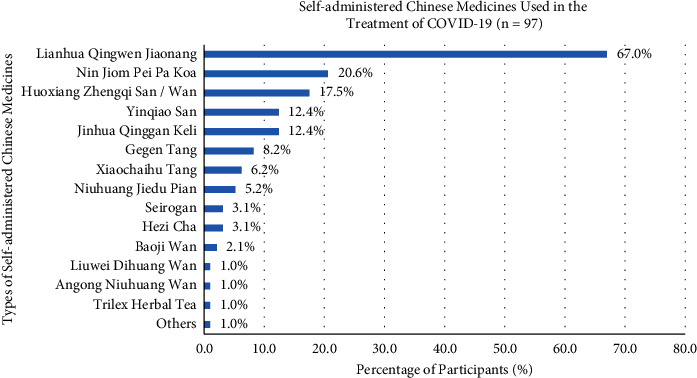
Participants' different types of self-administered Chinese medicines in the treatment of COVID-19.

**Figure 5:**
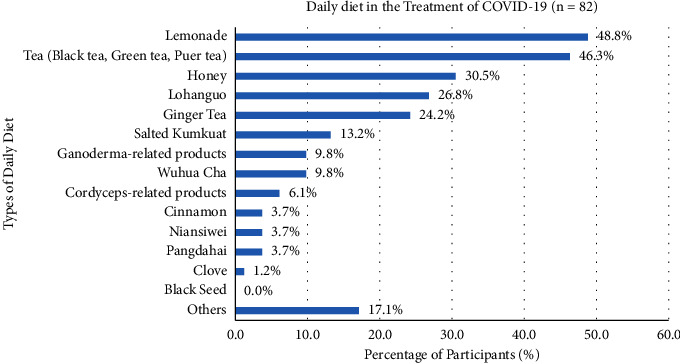
Participants' different types of daily diet in the treatment of COVID-19.

52.9% of participants used Chinese medicine in the treatment of COVID-19 in multiple ways, containing self-administered Chinese medicine (75.8%), unknown Chinese medicine practitioner prescription (52.3%), unknown folk prescription (29.7%), known Chinese medicine practitioner prescription (17.2%), and known folk prescription (9.4%) (Figure 3). Participants with different types of self-administered Chinese medicines in the treatment of COVID-19, such as Lianhua Qingwen Jiaonang (67.0%), Nin Jiom Pei Pa Koa (20.6%), Huoxiang Zhengqi San or Wan (17.5%), Yinqiao San (12.4%), Jinhua Qinggan Keli (12.4%), Gegen Tang (8.2%), Xiaochaihu Tang (6.2%), Niuhuang Jiedu Pian (5.2%), Seirogan (3.1%), Hezi Cha (3.1%), Baoji Wan (2.1%), Liuwei Dihuang Wan (1.0%), Angong Niuhuang Wan (1%), and Trilex Herbal Tea (1%) (Figure 4). Several *daily diets* for participants consisted of lemonade (48.8%), tea (e.g., black tea, green tea, and pu'er tea) (46.3%), honey (30.5%), lo han guo (26.8%), ginger tea (24.2%), salted kumquat (13.2%), Ganoderma-related products (9.8%), Wu Hua Cha (9.8%), Cordyceps-related products (6.1%), cinnamon (3.7%), NianSiWei (3.7%), Pangdahai (3.7%), and clove (1.2%), and 17.1% of participants chose the other types of diets (Figure 5).

**Figure 6:**
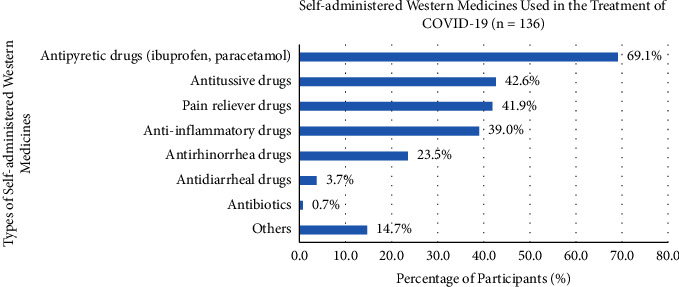
Participants' different types of self-administered Western medicines in the treatment of COVID-19.

There were around 61.9% of participants who used Western medicines in the treatment of COVID-19, containing known (54.0%) and unknown (46.0%) physician-prescribed Western medicines. The types of Western medicines used for participants involved antipyretic drugs (e.g., ibuprofen and paracetamol) (69.1%), antitussive drugs (42.6%), pain reliever drugs (41.9%), anti-inflammatory drugs (39.0%), antirhinorrhea drugs (23.5%), antidiarrheal drugs (3.7%), antibiotics (0.7%), and other Western medicines (14.7%) (Figure 6).

### 3.4. Part IV: Rehabilitation of COVID-19

**Figure 7:**
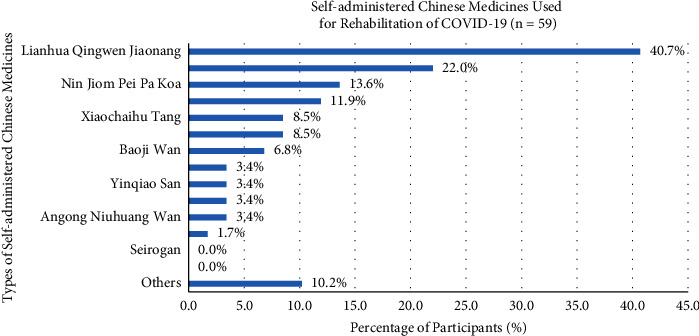
Participants' different types of self-administered Chinese medicines in the rehabilitation of COVID-19.

**Figure 8:**
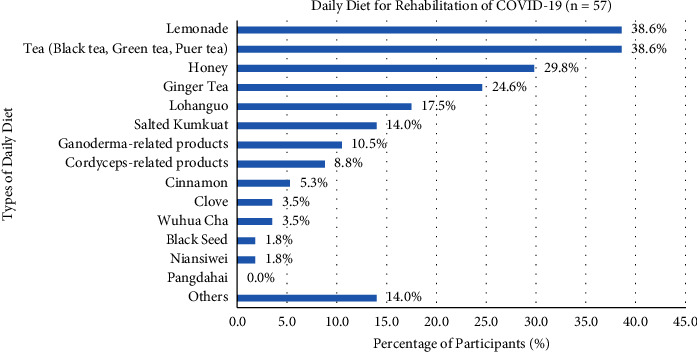
Participants' different types of daily diet in the rehabilitation of COVID-19.

Participants with nearly 52.4% used Chinese medicine in the rehabilitation of COVID-19, including unknown Chinese medicine practitioner prescription (68.5%), unknown folk prescriptions (3.6%), known Chinese medicine practitioner prescription (25.2%), and known folk prescriptions (2.7%). Self-administered Chinese medicines of participants in the rehabilitation of COVID-19 were, such as Lianhua Qingwen Jiaonang (40.7%), Huoxiang Zhengqi San or Wan (22.0%), Nin Jiom Pei Pa Koa (13.6%), Jinhua Qinggan Keli (11.9%), Xiaochaihu Tang (8.5%), Gegen Tang (8.5%), Baoji Wan (6.8%), Liuwei Dihuang Wan (3.4%), Yinqiao San (3.4%), Niuhuang Jiedu Pian (3.4%), Angong Niuhuang Wan (3.4%), Hezi Cha (1.7%), and others Chinese medicines (10.2%) (Figure 7). Some daily diets for participants consisted of lemonade (38.6%), tea (e.g. black tea, green tea, and pu'er tea) (38.6%), honey (29.8%), ginger Tea (24.6%), Lo Han Guo (17.5%), salted Kumquat (14.0%), Ganoderma-related products (10.5%), Cordyceps-related products (8.8%), cinnamon (5.3%), clove (3.5%), Wu Hua Cha (3.5%), black seed (1.8%), and NianSiWei (1.8%), and 14.0% of participants chose the other types of diets (Figure 8).

**Figure 9:**
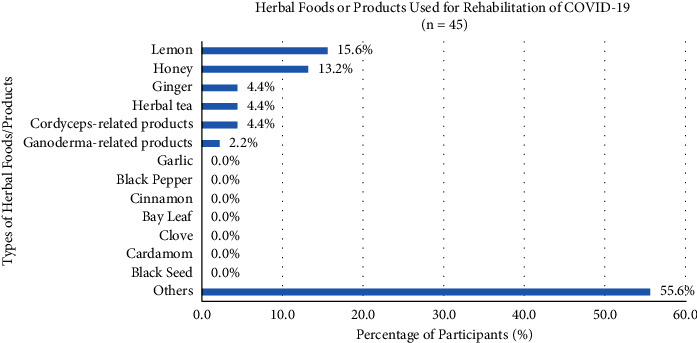
Participants' preferences for using herbal foods or products in the rehabilitation of COVID-19.

**Figure 10:**
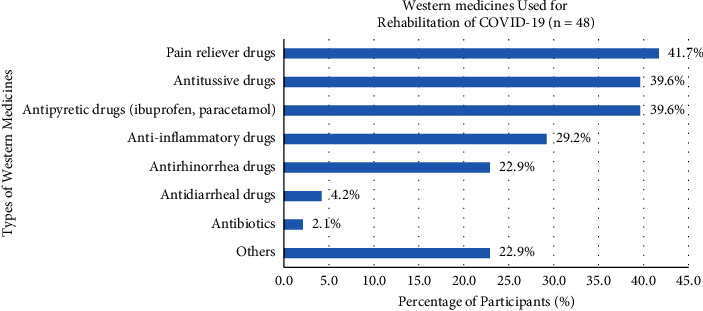
Use of Western medicines in the rehabilitation of COVID-19.

However, only 24.2% of participants would use herbal foods or products in the rehabilitation of COVID-19, which consisted of lemon (41.1%), honey (37.9%), ginger (27.6%), herbal tea (25.9%), Cordyceps-related products (15.5%), Ganoderma-related products (12.1%), garlic (8.6%), black pepper (6.9%), cinnamon (6.9%), bay leaf (5.2%), clove (5.2%), cardamom (3.4%), and other herbal foods or products (12.1%) (Figure 9). Besides, there were just 23.8% and 16.5% of participants who would use the medicinal soup and Western medicines in the rehabilitation of COVID-19, respectively. Meanwhile, 34.6% of participants used physician-prescribed Western medicines involving pain reliever drugs (41.7%), antitussive drugs (39.6%), antipyretic drugs (e.g., ibuprofen and paracetamol) (39.6%), anti-inflammatory drugs (29.2%), antirhinorrhea drugs (22.9%), antidiarrheal drugs (4.2%), antibiotics (2.1%), and other Western medicines (22.9%) (Figure 10).

### 3.5. Part V: Sources of Information for the Chinese Medicines or Western Medicines on the COVID-19

**Figure 11:**
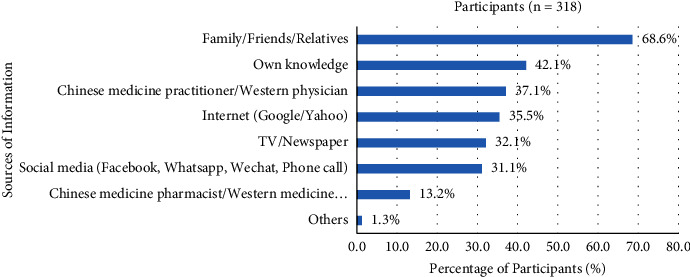
Sources of information for participants.

Participants received a piece of information about COVID-19, mainly from family (friends or relatives) (68.6%), own knowledge (42.1%), Chinese medicine practitioner, or Western physician (37.1%), Internet (Google or Yahoo) (35.5%), TV and newspaper (32.1%), social media (Facebook, WhatsApp, WeChat, and phone call) (31.1%), Chinese medicine pharmacist or Western medicine pharmacist (13.2%), and others (1.3%) (Figure 11).

### 3.6. Part VI: The Pattern of Chinese Medicines and Western Medicines' Usage in the Prevention, Treatment, and Rehabilitation of COVID-19

318 people participated in this survey, and only 311 were qualified, who used Chinese or Western medicines, respectively, during the prevention, treatment, and rehabilitation periods of COVID-19, based on (i) gender, (ii) age, and (iii) Chinese or Western medicines.

#### 3.6.1. Gender

311 people used Chinese medicine for the prevention, treatment, and rehabilitation periods of COVID-19. Its usage accounted for 128 males (41.2%) and 183 females (58.8%). A chi-square test was conducted and calculated obtaining *χ*^2^ (2, 311) frequency = 0.028 and *p*=0.986 (Table 2).

267 people used Western medicines for the prevention, treatment, and rehabilitation periods of COVID-19. Its usage accounted for 106 males (39.7%) and 161 females (60.3%). A chi-square test was conducted and calculated obtaining *χ*^2^ (2, 267) = 6.624 and *p*=0.036 (Table 2).

According to the post hoc test, the Bonferonni correction for the multiple comparisons of prevention, treatment, and rehabilitation by adjusted significant level to *α*' = 0.017. This showed the percentages of prevention, treatment, and rehabilitation for males and females with *χ*^2^ and *p* value.

34.9% of males and 52.8% of females were in the prevention stage, obtaining *χ*^2^ (1, 267) = 0.000 and *p* = 0.984 compared with treatment and rehabilitation stages (Table 3). 60.4% of males and 50.9% of females were in the treatment stage, obtaining *χ*^2^ (1, 267) = 2.577 and *p* = 0.108 compared with prevention and rehabilitation stages (Table 4). 5.0% of males and 13.7% of females were in the rehabilitation stage, obtaining *χ*^2^ (1, 267) = 6.234 and *p* = 0.013 compared with prevention and treatment stages (Table 5).

#### 3.6.2. Age

Chinese medicine usage accounting for 18 to 37 was 21.1%, 39.5%, and 39.5% of 147 people (47.3%); between 38 and 57 was 26.4%, 41.8%, and 31.8% of 110 people (35.4%); aged 58 years or above was 22.2%, 44.4%, and 33.3% of 54 (17.4%), respectively, for the prevention, treatment, and rehabilitation periods of COVID-19. A chi-square test was conducted and calculated obtaining *χ*^2^ (2, 331) = 2.168 and *p*=0.705 (Table 3).

Western medicine usage accounting for 18 to 37 was 32.1%, 56.6%, and 11.3% of 149 people (59.6%); between 38 and 57 was 41.2%, 49.4%, and 9.4% of 85 people (35.4%); aged 58 years or above was 30.4%, 60.9%, and 8.7% of 23 (8.6%), respectively, for the prevention, treatment, and rehabilitation periods of COVID-19. A chi-square test was conducted and calculated obtaining *χ*^2^ (2, 267) = 2.427 and *p*=0.658 (Table 3).

#### 3.6.3. Chinese or Western Medicines

People who used Chinese medicines for the prevention, treatment, and rehabilitation periods of COVID-19 were 23.2%, 41.2%, and 35.7%, respectively, whilst those who used Western medicines for the prevention, treatment, and rehabilitation periods of COVID-19 were 34.8%, 54.7%, and 10.5%. A chi-square test was conducted and calculated obtaining *χ*^2^ (2, 578) = 50.359 and *p* ≤ 0.001 (Table 4).

According to the post hoc test, the Bonferonni correction for the multiple comparisons of prevention, treatment, and rehabilitation by adjusted significant level to *α*' = 0.017. This showed the percentages of prevention, treatment, and rehabilitation for Chinese and Western medicines with *χ*^2^ and *p* value. 23.2% of males and 34.8% of females were in the prevention stage, obtaining *χ*^2^ (1, 578) = 9.609 and *p*=0.002 compared with treatment and rehabilitation stages. 41.2% of males and 54.7% of females were in the treatment stage, obtaining *χ*^2^ (1, 578) = 10.539 and *p*=0.001 compared with prevention and rehabilitation stages. 35.7% of males and 10.5% of females were in the rehabilitation stage, obtaining *χ*^2^ (1, 578) = 49.966 and *p* ≤ 0.001 compared with prevention and treatment stages (Table 6).

## 4. Discussion

This research has highlighted the behavioral prevention, treatment, and rehabilitation of using Western medicines or herbal products among the public in response to the Hong Kong COVID-19 pandemic.

The practice preventive measures against COVID-19 include wearing masks, using alcohol hand sanitizer, washing hands frequently, avoiding crowds, and staying home more often which were the personal hygiene monitoring according to the Hong Kong Government and World Health Organization (WHO) suggestions.

Wearing a mask was effective to prevent the spread of COVID-19 because it is primarily based on respiratory droplets and aerosols or close contact [20]. Alcohol hand sanitizer usually consisted of n-propanol, which was believed to damage the virus membrane and distribute its decoupling and protein synthesis. It was an anti-infectious agent for killing pathogens in the hands [21].

Washing hands frequently might stop the spread of COVID-19 with soap and running water, which were the critical importance for removing a virus or pathogen within the hands, and preventing the droplets from the infected person or direct contact with the contaminated materials [22].

Avoiding crowds and social distance decreased an individual's likelihood of contracting COVID-19 by reducing its transmission through person-to-person interaction, especially in preventing the social community outbreak [23].

Staying home more often or working at home minimized contact with others, reducing the spread of SARS-CoV-2 among participants and the risk of infection [24]. Thus, nearly a hundred percent of participants wore a mask, and above eighty percent of participants used alcohol hand sanitizer as well as washed hands frequently which were the common practice preventive measures against COVID-19.

Only a few participants (9.1%) were taking herbal food or products for the prevention of COVID-19 compared with Western medicines or supplements (15.7%). More than 61.6% of participants did not take any herbal food or Western medicines. Since the specific drugs and vaccines were not yet to be developed, taking herbal food or products might be an alternative COVID-19 preventive therapies [25]. Some participants with COVID-19 would be recovered without needing to go to the hospital or take any herbal food and Western medicines [26].

Western medicine or vitamin supplements, such as C, D, B, multivitamins (86.9%), and zinc (14.8%) were frequently chosen by participants because these might have a beneficial effect, potentially reducing SARS-CoV-2 viral load and length of hospitalization, which enhanced the immune function and reduced the risk of COVID-19 infection [27], whilst herbal foods or products included herbal soup (66.4%), and herbal tea (39.4%) was in a higher percentage for the participants' selection. Yi Mi Fang Feng and Wu Shen Tang were the traditional Chinese herbal soups that detoxified the lung and enhanced the immune system, relieving upper respiratory tract infections by dispelling wind and cold [28].

52.9% of participants used Chinese medicine to treat COVID-19, and 41.1% of them were self-administered Chinese medicine and did not know the Chinese medicine practitioner's prescription. Lianhua Qingwen Jiaonang (67.0%) was the most common one for self-administered Chinese medicine. In the previous research, Lianhua Qingwen Jiaonang possessed antiviral and anti-inflammatory actions that inhibited the SARS-CoV-2 virus replication and reduced viral content in the cytomembrane and cytoplasm, as well as suppressed cytokine overactivation. This significantly ameliorated the symptoms of cough, fever, and fatigue, which increased the effective rate and shortened the recovery rate in patients [29]. Other Chinese medicines such as Nin Jiom Pei Pa Koa (20.6%), Huoxiang Zhengqi San or Wan (17.5%), Yinqiao San (12.4%), and Jinhua Qinggan Keli (12.4%) were lower in percentage usage in contrast to Lianhua Qingwen Jiaonang, which used to regulate the effects on an immune system.

Besides, a daily diet was another important part of treating COVID-19. Lemonade (48.8%), tea (e.g., black tea, green tea, and pu'er tea) (46.3%), and honey (30.5%) were the most popular. This was not surprising that 48.8% of participants selected lemonade as a daily diet. It consisted of ascorbic acid for the body's health but no evidence could “boost” or “supercharge” an immune system to prevent infections and treatment of COVID-19 [30]. Black tea, green tea, and pu'er tea contained polyphenols that could inhibit the antiviral activities against positive-sense single-stranded RNA virus (SARS-CoV-2) for the treatment of COVID-19 [31]. Honey was a neutral therapy because of its ability to attenuate acute inflammation by enhancing immune response. It also improved the comorbid conditions and antiviral activities of an enveloped virus SARS-CoV-2 for patients with COVID-19 [32].

The usage of Western medicine was around 61.9%, which was a little bit higher than Chinese medicine, and half of the participants (54%) were known the physician-prescribed Western medicines. Antipyretic drugs (e.g., ibuprofen and paracetamol) (69.1%), antitussive drugs (42.6%), pain reliever drugs (41.9%), and anti-inflammatory drugs (39.0%) were the most commonly used for the treatment of COVID-19. Since antipyretic drugs such as ibuprofen and acetaminophen were usually taken by individuals to reduce the discomfort of fever and to shorten the duration of viral illness, ibuprofen demonstrated superior efficacy in fever reduction compared to paracetamol in the previous study [33]. The antitussive drugs appeased effects on the lung and respiratory infections that influenced the binding affinity for the main protease (Mpro) of SARS-CoV-2, as an anti-viral agent for the treatment of COVID-19 [34]. Pain reliever drugs were required to reduce pain, as the COVID-19 infection was associated with myalgias, referred pain, and widespread hyperalgesia [35]. Because SARS-CoV-2 was a rapid self-replication virus, a large number of inflammatory for cell infiltration led to acute lung injury, acute respiratory distress syndrome (ARDS), and death. Anti-inflammatory drugs were effective for altering susceptibility to infection and modifying the expression of angiotensin-converting enzyme 2 (ACE2), as well as the cell entry receptor for SARS-CoV-2. This also modulated the replication of SARS-CoV-2 in host cells and enhanced the immune response to SARS-CoV-2 for the treatment of COVID-19 [36].

There were a high proportion of participants who used Chinese medicine for the rehabilitation of COVID-19. However, half of the participants (50%) were unknown of the Chinese medicine practitioner's prescription. The usage of Lianhua Qingwen Jiaonang (40.7%) served as a self-administered Chinese medicine in the rehabilitation stage and was still at a high percentage, which is the same as the treatment period. Huoxiang Zhengqi San or Wan was the second highest proportion and was different from the treatment period using Nin Jiom Pei Pa Koa (13.6%). Huoxiang Zhengqi San or Wan formula treated gastrointestinal-type colds and improved clinical symptoms, as well as patient prognosis, which could pave a complementary medicine for rehabilitation [37]. The daily diet in rehabilitation was alike to the treatment, lemonade (38.6%), tea (e.g., black tea, green tea, and pu'er tea) (38.6%), and honey (29.8%), as well as ginger tea (24.6%) first appeared. The ginger tea possessed an anti-inflammatory effect and ameliorative effect in musculoskeletal and rheumatism patients by inhibiting cyclooxygenase and lipoxygenase pathway in synovial fluid during the rehabilitation period [38].

Comparatively, only 24.2% of participants would use herbal foods or products in the rehabilitation of COVID-19, which consisted of lemon (41.1%), honey (37.9%), ginger (27.6%), and herbal tea (25.9%), and discussed the prevention and treatment before. There were other herbal foods or products such as Cordyceps-related products (15.5%) and Ganoderma-related products (12.1%). Its bioactive constituents produced the interleukin, such as (IL)-1*β*, IL-2, IL-6, IL-8, IL-10, and IL-12, and tumor necrosis factor (TNF)-*α*, phagocytosis stimulation of immune cells, and nitric oxide production for the stimulation of inflammatory response via mitogen-activated protein kinase pathway to modulate or enhance the functions of an immune system [39].

The percentage of participants who used *medicinal soup* (23.8%) in the rehabilitation was much greater than that of the Western medicines (16.5%) because some medicinal soups were suitably used in either the prevention or rehabilitation of COVID-19. In contrast to the Western medicines for the treatment stage, there was a different percentage between pain reliever drugs (41.7%), antitussive drugs (39.6%), antipyretic drugs (e.g., ibuprofen and paracetamol) (39.6%), and anti-inflammatory drugs (29.2%). This percentage of Western drugs indicated that participants were more required to kill the myalgias, referred pain, and hyperalgesia during the rehabilitation period.

Participants received information about Chinese medicines or Western medicines for COVID-19 from family, friends, and relatives mainly. These were the primary sources, which directly affected the personal feeling and played an important role in immediate action flight against COVID-19. Around 40% of participants believed in their knowledge to decide the usage of Chinese medicines or Western medicines during COVID-19, and there existed a risk of the wrong selection.

Technology was changing with each passing day, and the sources of information also come from the Internet, TV or newspaper, and social media. Participants received the wrong message because of spreading misinformation surrounding COVID-19 [40]. Only a few participants listen to and follow the instructions from the Chinese medicine practitioner or Western physician and Chinese medicine pharmacist or Western medicine pharmacist, as a result, decreased the recovery rate and increased the death rate from COVID-19. Thus, participants' sociodemographic background was related to their manner of the sources of information received for the Chinese medicines or Western medicines of COVID-19.

Three hypotheses were stated: Do (i) gender or (ii) age affect the pattern of Chinese medicines and Western medicines' usage in the prevention, treatment, and rehabilitation of COVID-19? (iii) Can Chinese medicines and Western medicines' usage affect the prevention, treatment, and rehabilitation of COVID-19? (i) No significant association between gender when using Chinese medicine, but Western medicine with a significant association (Table 2) based on the post hoc test for Bonferonni correction, multiple comparisons between prevention, treatment, and rehabilitation stages for COVID-19. It showed the *p* values of prevention (0.984) (Table 5) and treatment (0.108) (Table 5) were insignificant that were higher than *α* = 0.05, and only the *p* value of rehabilitation (0.013) was significant. This indicated females with a high proportion used Western medicines than males in the rehabilitation stage (Table 5). (ii) No significant association between age either in Chinese medicine or Western medicine usage for COVID-19 (Table 3). (iii) It also indicated that there was no significant association between the Chinese and Western medicines used for COVID-19 (Table 4). However, the *p* values were significant for the three combinations when the Bonferonni correction was applied with an adjusted significant level (*α*' = 0.017) (Table 6). Surprisingly, participants used Chinese medicines for the rehabilitation of COVID-19 triple that of Western medicines (10.5%). It may be Chinese medicines for reinvigoration (*Guben Peiyuan*) within the body's functions during the rehabilitation stage, such as Lianhua Qingwen Jiaonang and Huoxiang Zhengqi San or Wan, which were also used to prevent or treat long-term COVID-19.

## 5. Limitations

The data collection for the questionnaire relied on the website. This may have data redundancy and affect the data accuracy as the survey was anonymous. Some participants did not know the data entry system and give misleading information. Besides, the sample size of this survey was a little limited; it cannot represent the whole population in Hong Kong using Chinese and Western medicines or herbal products during the prevention, treatment, and rehabilitation periods. This was only a small population investigation.

## 6. Conclusion

The original article concluded that sociodemographic information related to personal behavior prevention. Generally, participants with good behavior measure for preventing COVID-19. Several Western medicines or herbal products, daily diets, as well as medicinal soups were used for the treatment and rehabilitation of COVID-19. Some participants used an unknown Chinese medicine practitioner's prescription, and self-administered Chinese medicine was the most common. Participants were unknown with the physician-prescribed Western medicines, and it might appear as medicine abuse without a practitioner's prescription. The pattern of Chinese medicines and Western medicines' usage in the prevention, treatment, and rehabilitation of COVID-19 was the core part; it has shown the statistically significant association between the variables according to gender, age, and Chinese or Western medicines. The topic of this article was just the beginning of an investigation; it required further development of the Chinese medicines with herbal-drug interaction for participants to fight against COVID-19 based on the abovementioned pattern of the prevention, treatment, and rehabilitation periods.

## Figures and Tables

**Table 1:** Sociodemographic information for participants.

	Participants (*N*) (%)	Participants (*N*)
*(1) Gender (n = 318)*		
Male	45.6	145
Female	54.4	173
*(2) Age (n = 318)*		
18–27	15.7	50
28–37	37.4	119
38–47	15.7	50
48–57	15.4	49
58–67	10.4	33
68–77	4.1	13
>78	1.3	4
*(3) Educational level (n = 318)*		
Primary school or below	5.0	16
Secondary school	32.4	103
High diploma or associate degree	15.7	50
Degree	34.3	109
Master's degree	10.4	33
Doctoral degree	2.2	7
*(4) Place of residence (n = 318)*		
Hong Kong Island	10.7	34
Kowloon	27.0	86
New territories	58.2	185
Islands District	2.8	9
Unknown	1.3	4
*(5) Presence of chronic illness (n = 318)*		
Yes	19.2	61
No	80.8	257
*(6) Examples of chronic illnesses (n = 68)*		
Musculoskeletal	1.6	1
Respiratory	3.3	2
Gout	3.3	2
Cancer	8.2	5
Cardiovascular	9.8	6
Diabetes	18.1	11
Others	55.7	34

**Table 2:** Chinese medicines and Western medicines used for COVID-19 depend on gender.

	Prevention	Treatment	Rehabilitation	Row total
*Chinese medicines*				
Male				
Frequency (expected) (*χ*^2^)	30 (29.6) (0.00)	53 (52.7) (0.00)	45 (45.7) (0.01)	128
Row (%)	23.4%	41.4%	35.2%	41.2%
Column (%)	41.7%	41.4%	40.5%	
Female				
Frequency (expected) (*χ*^2^)	42 (42.4) (0.00)	75 (75.3) (0.00)	66 (65.3) (0.01)	183
Row (%)	23.0%	41.0%	36.1%	58.8%
Column (%)	58.3%	58.6%	59.5%	
Column total	72	128	111	311
	23.2%	41.2%	35.7%	100%
Chi-square test	*χ* ^2^: 0.028			—
	DF: 2			
	*p* value: 0.986			

*Western medicines*				
Male				
Frequency (expected) (*χ*^2^)	37 (36.9) (0.00)	64 (58.0) (0.63)	5 (11.1) (3.37)	106
Row (%)	34.9%	60.4%	4.7%	39.7%
Column (%)	39.8%	43.8%	17.9%	
Female				
Frequency (expected) (*χ*^2^)	56 (56.1) (0.00)	82 (88.0) (0.41)	23 (16.9) (2.22)	161
Row (%)	34.8%	50.9%	14.3%	60.3%
Column (%)	60.2%	56.2%	82.1%	
Column total	93	146	28	267
	34.8%	54.7%	10.5%	100%
Chi-square test	*χ* ^2^: 6.624			—
	DF: 2			
	*p* value: 0.036			

**Table 3:** Chinese medicines or Western medicines used for COVID-19 depend on age.

	Prevention	Treatment	Rehabilitation	Row total
*Chinese medicines*				
18–37				
Frequency (expected) (*χ*^2^)	31 (34.0) (0.27)	58 (60.5) (0.10)	58 (52.5) (0.58)	147
Row (%)	21.1%	39.5%	39.5%	47.3%
Column (%)	43.1%	45.3%	52.3%	
38–57				
Frequency (expected) (*χ*^2^)	29 (25.5) (0.49)	46 (45.27) (0.01)	35 (39.3) (0.46)	110
Row (%)	26.4%	41.8%	31.8%	35.4%
Column (%)	40.3%	35.9%	31.5%	
58 or above				
Frequency (expected) (*χ*^2^)	12 (12.5) (0.02)	24 (22.2) (0.14)	18 (19.27) (0.08)	54
Row (%)	22.2%	44.4%	33.3%	17.4%
Column (%)	16.7%	18.8%	16.2%	
Column total	72	128	111	311
	23.2%	41.2%	35.7%	100%
Chi-square test	*χ* ^2^: 2.168			—
	DF: 4			
	*p* value: 0.705			

*Western medicines*				
18–37				
Frequency (expected) (*χ*^2^)	51 (55.4) (0.35)	90 (86.9) (0.11)	18 (16.7) (0.11)	159
Row (%)	32.1%	56.6%	11.3%	59.6%
Column (%)	54.8%	61.6%	64.3%	
38–57				
Frequency (expected) (*χ*^2^)	35 (29.5) (0.98)	42 (46.5) (0.43)	8 (8.91) (0.09)	85
Row (%)	41.2%	49.4%	9.4%	31.8%
Column (%)	37.6%	28.8%	28.6%	
58 or above				
Frequency (expected) (*χ*^2^)	7 (8.01) (0.13)	14 (12.6) (0.16)	2 (2.41) (0.07)	23
Row (%)	30.4%	60.9%	8.7%	8.6%
Column (%)	7.5%	9.6%	7.1%	
Column total	93	146	28	267
	34.8%	54.7%	10.5%	100%
Chi-square test	*χ* ^2^: 2.427			—
	DF: 4			
	*p* value: 0.658			

**Table 4:** Chinese or Western medicines used in the prevention, treatment, and rehabilitation of COVID-19.

	Prevention	Treatment	Rehabilitation	Row total
*Chinese medicines*				
Frequency (expected) (*χ*^2^)	72 (88.8) (3.17)	128 (147.4) (2.56)	111 (74.8) (17.53)	311
Row (%)	23.2%	41.2%	35.7%	53.8%
Column (%)	43.6%	46.7%	79.9%	

*Western medicines*				
Frequency (expected) (*χ*^2^)	93 (76.2) (3.69)	146 (126.6) (2.98)	28 (64.2) (20.4)	267
Row (%)	34.8%	54.7%	10.5%	46.2%
Column (%)	56.4%	53.3%	20.1%	
Column total	165	274	139	578
	28.5%	47.4%	24.0%	100%
Chi-square test	*χ* ^2^: 50.359			—
	DF: 2			
	*p* value: ≤0.001			

**Table 5:** Bonferonni correction for the multiple comparisons among prevention, treatment, and rehabilitation stages for COVID-19.

	Participants (*N*)	Others	Participants (*N*; %)
*Prevention*			
Male	37	69	34.9%
Female	56	105	52.8%
Rehabilitation and treatment compared with prevention: *χ*^2^ (1,267) = 0.000 and *p*=0.984			

*Treatment*			
Male	64	41	60.4%
Female	82	79	50.9%
Prevention and rehabilitation compared with treatment: *χ*^2^ (1,267) = 2.577 and *p*=0.108			

*Rehabilitation*			
Male	5	101	5.0%
Female	23	138	13.7%
Prevention and treatment compared with rehabilitation: *χ*^2^ (1,267) = 6.234 and *p*=0.013			

**Table 6:** Bonferonni correction for the Chinese or Western medicines used in the prevention, treatment, and rehabilitation stages for COVID-19.

	Prevention (*N*)	Others	Prevention (%)
*Prevention*			
Chinese medicines	72	239	23.2%
Western medicines	93	174	34.8%
Rehabilitation and treatment compared with prevention: *χ*^2^ (1,578) = 9.609 and *p*=0.002			

*Treatment*			
Chinese medicines	128	183	41.2%
Western medicines	146	121	54.7%
Prevention and rehabilitation compared with treatment: *χ*^2^ (1,578) = 10.539 and *p*=0.001			

*Rehabilitation*			
Chinese medicines	111	200	35.7%
Western medicines	28	239	10.5%
Prevention and treatment compared with rehabilitation: *χ*^2^ (1, 578) = 49.966 and *p* ≤ 0.001			

## Data Availability

The data used to support the findings of this study are available from the corresponding author on request.

## References

[1] Peng P., Ho P. L., Hota S. S. (2020). Outbreak of a new coronavirus: what anaesthetists should know. *British Journal of Anaesthesia*.

[2] Law S., Leung A. W., Xu C. (2020). Severe acute respiratory syndrome (SARS) and coronavirus disease-2019 (COVID-19): from causes to preventions in Hong Kong. *International Journal of Infectious Diseases*.

[3] World Health Organization (2019). WHO director-general’s statement on ihr emergency committee on novel coronavirus (2019-nCoV). https://www.who.int/director-general/speeches/detail/who-director-general-s-statement-on-ihr-emergency-committee-onnovel-coronavirus.

[4] Cascella M., Rajnik M., Aleem A., Dulebohn S. C., Di Napoli R. (2020). Features, evaluation, and treatment of coronavirus (COVID-19). *StatPearls*.

[5] World Health Organization (2022). WHO coronavirus (COVID-19) dashboard. https://covid19.who.int/.

[6] Lam H. Y., Lam T. S., Wong C. H. (2020). The epidemiology of COVID-19 cases and the successful containment strategy in Hong Kong-January to May 2020. *International Journal of Infectious Diseases*.

[7] Centre of Health Protection (2019). Countries/areas with reported cases of Coronavirus Disease-2019 (COVID-19). https://www.chp.gov.hk/files/pdf/statistics_of_the_cases_novel_coronavirus_infection_en.pdf.

[8] Wong S., Kwok K. O., Chan F. (2020). What can countries learn from Hong Kong’s response to the COVID-19 pandemic?. *Canadian Medical Association Journal*.

[9] Wong S., Tan D., Zhang Y. (2021). A tale of 3 asian cities: how is primary care responding to COVID-19 in Hong Kong, Singapore, and beijing. *The Annals of Family Medicine*.

[10] Cheng V. C., Wong S. C., Tong D. W. (2022). Multipronged infection control strategy to achieve zero nosocomial coronavirus disease 2019 (COVID-19) cases among Hong Kong healthcare workers in the first 300 days of the pandemic. *Infection Control and Hospital Epidemiology*.

[11] Hung K. K., Walline J. H., Chan E. (2022). Health service utilization in Hong Kong during the COVID-19 pandemic-A cross-sectional public survey. *International Journal of Health Policy and Management*.

[12] World Health Organization (2019). Coronavirus disease (COVID-19) advice for the public. https://www.who.int/emergencies/diseases/novel-coronavirus-2019/advice-for-public.

[13] Nilashi M., Samad S., Yusuf S., Akbari E. (2020). Can complementary and alternative medicines be beneficial in the treatment of COVID-19 through improving immune system function?. *Journal of Infection and Public Health*.

[14] Pandolfi S., Simonetti V., Ricevuti G., Chirumbolo S. (2021). Paracetamol in the home treatment of early COVID-19 symptoms: a possible foe rather than a friend for elderly patients?. *Journal of Medical Virology*.

[15] Chavda V. P., Patel A. B., Vihol D. (2022). Herbal remedies, nutraceuticals, and dietary supplements for COVID-19 management: an update. *Clinical Complementary Medicine and Pharmacology*.

[16] Feng Z., Yang J., Xu M. (2021). Dietary supplements and herbal medicine for COVID-19: a systematic review of randomized control trials. *Clinical nutrition ESPEN*.

[17] Quincho-Lopez A., Benites-Ibarra C. A., Hilario-Gomez M. M., Quijano-Escate R., Taype-Rondan A. (2021). Self-medication practices to prevent or manage COVID-19: a systematic review. *PLoS One*.

[18] Ahmed I., Hasan M., Akter R. (2020). Behavioral preventive measures and the use of medicines and herbal products among the public in response to Covid-19 in Bangladesh: a cross-sectional study. *PLoS One*.

[19] Zhang W., Deng H. (2021). A survey of clinical evidence evaluation systems for traditional Chinese medicine. *Evidence-based Complementary and Alternative Medicine*.

[20] Law S. K., Leung A. W. N., Xu C. (2020). Are face masks useful for limiting the spread of COVID-19?. *Hong Kong Medical Journal*.

[21] Prajapati P., Desai H., Chandarana C. (2022). Hand sanitizers as a preventive measure in COVID-19 pandemic, its characteristics, and harmful effects: a review. *Journal of the Egyptian Public Health Association*.

[22] Beiu C., Mihai M., Popa L., Cima L., Popescu M. N. (2020). Frequent hand washing for COVID-19 prevention can cause hand dermatitis: management tips. *Cureus*.

[23] Fazio R. H., Ruisch B. C., Moore C. A., Granados Samayoa J. A., Boggs S. T., Ladanyi J. T. (2021). Social distancing decreases an individual’s likelihood of contracting COVID-19. *Proceedings of the National Academy of Sciences of the U S A*.

[24] Birimoglu Okuyan C., Begen M. A. (2022). Working from home during the COVID-19 pandemic, its effects on health, and recommendations: the pandemic and beyond. *Perspectives in Psychiatric Care*.

[25] Panyod S., Ho C. T., Sheen L. Y. (2020). Dietary therapy and herbal medicine for COVID-19 prevention: a review and perspective. *Journal of Traditional and Complementary Medicine*.

[26] Singhal T. (2020). A review of coronavirus disease-2019 (COVID-19). *Indian J Pediatr*.

[27] Gombart A. F., Pierre A., Maggini S. (2020). A review of micronutrients and the immune system-working in harmony to reduce the risk of infection. *Nutrients*.

[28] Law S., Xu C., Leung A. W. N. (2020). Use of Chinese medicine in the prevention and treatment of COVID-19 in China and Asia. *Asian Education and Development Studies*.

[29] Fan S. J., Liao J. K., Wei L., Wang B. Y., Kai L., Tan D. X. (2022). Treatment efficacy of Lianhua Qingwen capsules for eraly-stage COVID-19. *Am J Transl Res*.

[30] Xi S., Li Y., Yue L. (2020). Role of traditional Chinese medicine in the management of viral pneumonia. *Frontiers in Pharmacology*.

[31] National Academies (2019). The acid in lemon juice will not kill coronaviruses in your body. https://www.nationalacademies.org/based-on-science/lemon-juice-does-not-cure-covid-19.

[32] Mhatre S., Srivastava T., Naik S., Patravale V. (2021). Antiviral activity of green tea and black tea polyphenols in prophylaxis and treatment of COVID-19: a review. *Phytomedicine*.

[33] Hossain K. S., Hossain M. G., Moni A. (2020). Prospects of honey in fighting against COVID-19: pharmacological insights and therapeutic promises. *Heliyon*.

[34] Jamerson B. D., Haryadi T. H. (2020). The use of ibuprofen to treat fever in COVID-19: a possible indirect association with worse outcome?. *Medical Hypotheses*.

[35] Kumar N., Awasthi A., Kumari A. (2022). Antitussive noscapine and antiviral drug conjugates as arsenal against COVID-19: a comprehensive chemoinformatics analysis. *Journal of Biomolecular Structure and Dynamics*.

[36] El-Tallawy S. N., Nalamasu R., Pergolizzi J. V., Gharibo C. (2020). Pain management during the COVID-19 pandemic. *Pain Ther*.

[37] Chen J. S., Alfajaro M. M., Chow R. D. (2021). Non-steroidal anti-inflammatory drugs dampen the cytokine and antibody response to SARS-CoV-2 infection. *Journal of Virology*.

[38] Xiao M., Tian J., Zhou Y. (2020). Efficacy of Huoxiang Zhengqi dropping pills and Lianhua Qingwen granules in treatment of COVID-19: a randomized controlled trial. *Pharmacological Research*.

[39] Oladele J. O., Ajayi E. I., Oyeleke O. M. (2020). A systematic review on COVID-19 pandemic with special emphasis on curative potentials of Nigeria based medicinal plants. *Heliyon*.

[40] Zarocostas J. (2020). How to fight an infodemic. *The Lancet*.

